# Complete genome sequence of *Actinomyces oris* strain MG-1: an oral commensal critical for dental plaque formation

**DOI:** 10.1128/mra.00694-25

**Published:** 2025-07-31

**Authors:** Bibek G. C., Kexin Tan, Chenggang Wu

**Affiliations:** 1 Department of Microbiology & Molecular Genetics, The University of Texas Health Science Center 12340, Houston, Texas, USA; Queens College Department of Biology, Queens, New York, USA

**Keywords:** *Actinomyces oris*, dental plaque, genome sequencing, fimbriae

## Abstract

*Actinomyces oris* MG-1 is a key oral commensal involved in dental plaque formation. We report its complete genome sequence, generated using Illumina and Oxford Nanopore sequencing. The genome consists of a single circular chromosome of 3,042,829 bp, providing a resource for future functional studies.

## ANNOUNCEMENT

*Actinomyces oris* is a prominent early colonizer in oral biofilms and plays a central role in dental plaque formation, largely due to its surface-associated fimbrial structures (1). Strain MG-1, acquired from ATCC (#43146) and maintained in our laboratory, has been the principal model for studying *A. oris* fimbriae and coaggregation. It was originally isolated from dental plaque and initially designated as *A. viscosus* (2), reclassified as *A. naeslundii* (3), and later recognized as *A. oris* (4). Although a draft genome sequence is available (BioProject PRJNA327886), it consists of 485 contigs and lacks the completeness required for detailed genomic and functional analyses.

Here, we report the complete genome of *A. oris* MG-1, obtained using a hybrid sequencing approach that combines both short-read Illumina and long-read Oxford Nanopore Technologies (ONT) platforms. The strain was revived from a –80°C glycerol stock by streaking on heart infusion agar and incubating at 37°C in a 5% CO_₂_ atmosphere for 48 h. Colonies were harvested, and genomic DNA was extracted using the ZymoBIOMICS DNA Miniprep Kit (Zymo Research, D4300), following the manufacturer’s protocol. DNA quantity was measured with a Qubit 4 fluorometer (Thermo Fisher Scientific, Q33327). Sequencing was performed by SeqCenter (Pittsburgh, PA) using both Illumina and ONT platforms. Illumina libraries were prepared using the tagmentation-based Illumina DNA Prep kit with custom 10 bp unique dual indices, targeting a 280 bp insert size. Paired-end (2 × 151 bp) sequencing was conducted on a NovaSeq X Plus instrument. Raw reads were demultiplexed, adapter-trimmed, and quality-filtered using *bcl-convert* v4.2.4, yielding 6,538,956 reads totaling 887 Mbp with >Q30 quality, 50x coverage. Default parameters were used for all software unless otherwise specified. ONT libraries were prepared with the PCR-free Ligation Sequencing Kit (SQK-NBD114.96) and NEBNext Companion Module (E7180L), without additional fragmentation or size selection. Sequencing was performed on a GridION platform using R10.4.1 flow cells in 400 bp mode. Dorado v0.5.3 was used for basecalling under the super-accurate (sup) and 5mC/5hmC models. FASTQ files were extracted from BAM files using *samtools fastq* v1.20. ONT sequencing produced 269,129 reads totaling ~907 Mbp with an N50 read length of 8,690 bp. Residual adapter sequences were trimmed with *Porechop*. *De novo* assembly was performed using *Flye* v2.9 with the nano-hq model, integrating both Illumina and ONT reads (5). Assembly quality was assessed with *QUAST* v5.0.2 (6). Genome circularization was confirmed using Circlator v1.5.5 (7). Genome annotation was performed with the NCBI Prokaryotic Genome Annotation Pipeline (PGAP) v5.2 (8). QUAST analysis confirmed a high-quality assembly: a single circular contig of 3,042,829 bp with an N50 equal to the full genome length, a GC content of 68.47%, and no ambiguous bases (0 Ns per 100 kbp). The final sequencing depth was approximately 50×. PGAP predicted 2,446 protein-coding genes, 51 tRNAs, and 9 rRNAs. The genome was not rotated after circularization.

This complete genome sequence of *A. oris* MG-1 establishes a robust foundation for future investigations into fimbrial structure–function relationships and microbial interactions, particularly with *Fusobacterium nucleatum*.

## Data Availability

The complete genome sequence of *Actinomyces oris* MG-1 has been deposited in GenBank under accession number CP194891. Raw sequence data for Illumina and ONT reads are available in the Sequence Read Archive (SRA) under BioProject accession number PRJNA1271997.
